# Supplementary material for: Enrichment of polycyclic aromatic hydrocarbon metabolizing microorganisms on the oral mucosa of tobacco users
Source: PeerJ. 2024 Jan 3;12:e16626. doi: 10.7717/peerj.16626 (PMC10771095; doi:10.7717/peerj.16626)
Supplement: Supplemental Information 1 [file peerj-12-16626-s001.pdf]

Supplemental Data

Enrichment of polycyclic aromatic hydrocarbon metabolizing microorganisms in tobacco smokers' mouths

Lin Tao, M. Paul Chiarelli, Sylvia I. Pavlova, Antonia Kolokythas, Joel L Schwartz , James V. DeFrancesco, Benjamin Salameh, Stefan J. Green, Guy R. Adami

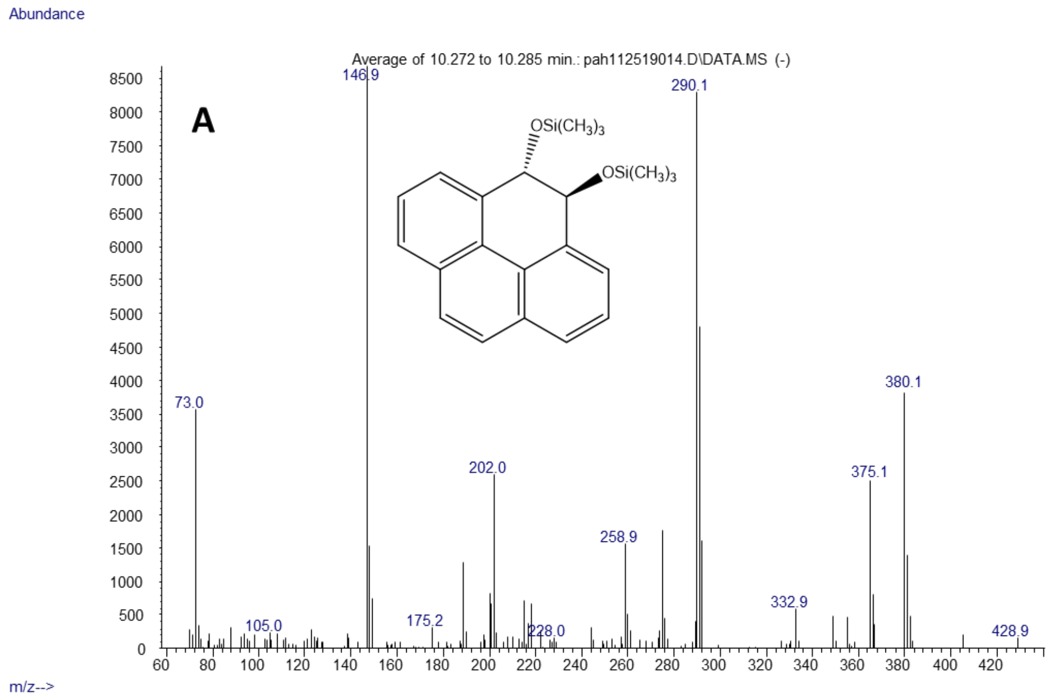

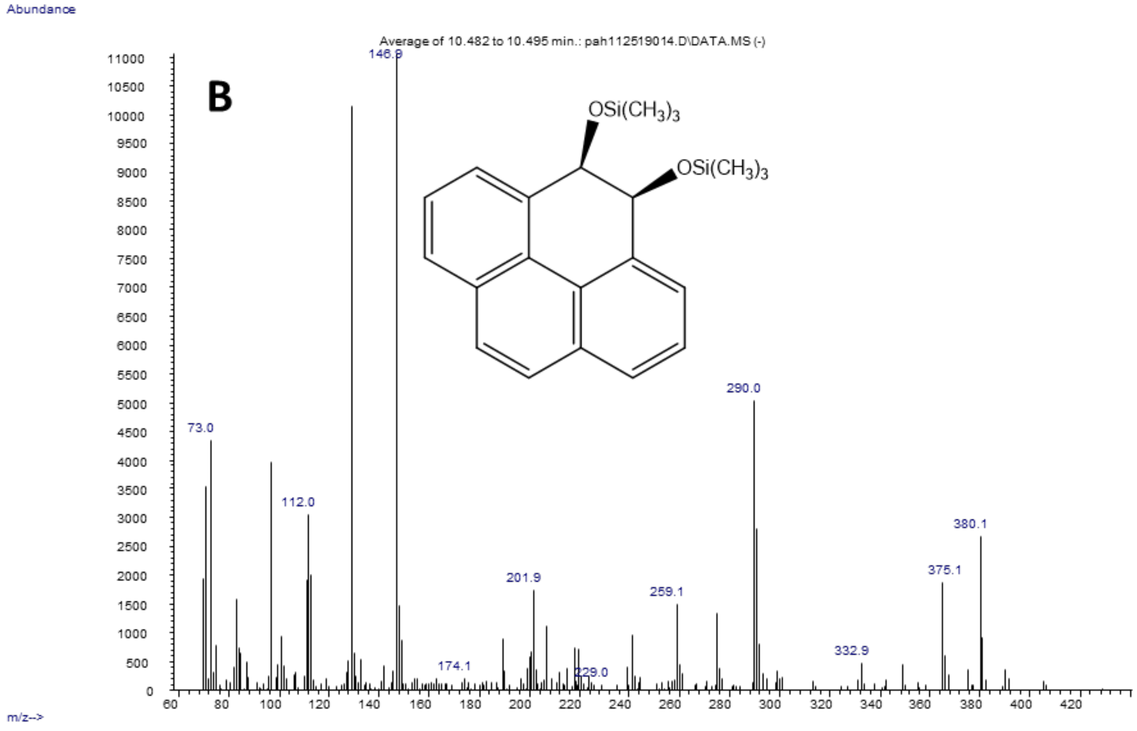

Supplemental Figure 1: Electron ionization mass spectra of compounds suggested to be **A)** cis- and **B)** trans-4,5-dihydroxy-4,5-dihydropyrene from incubation of positive control *Mycobacterium rutilum* with pyrene

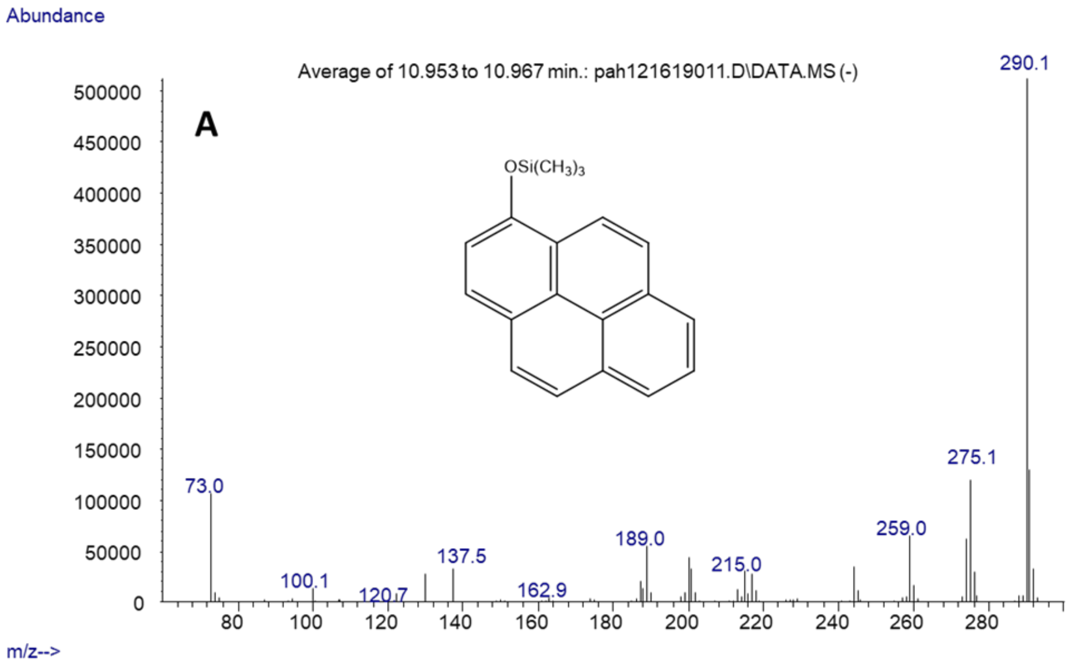

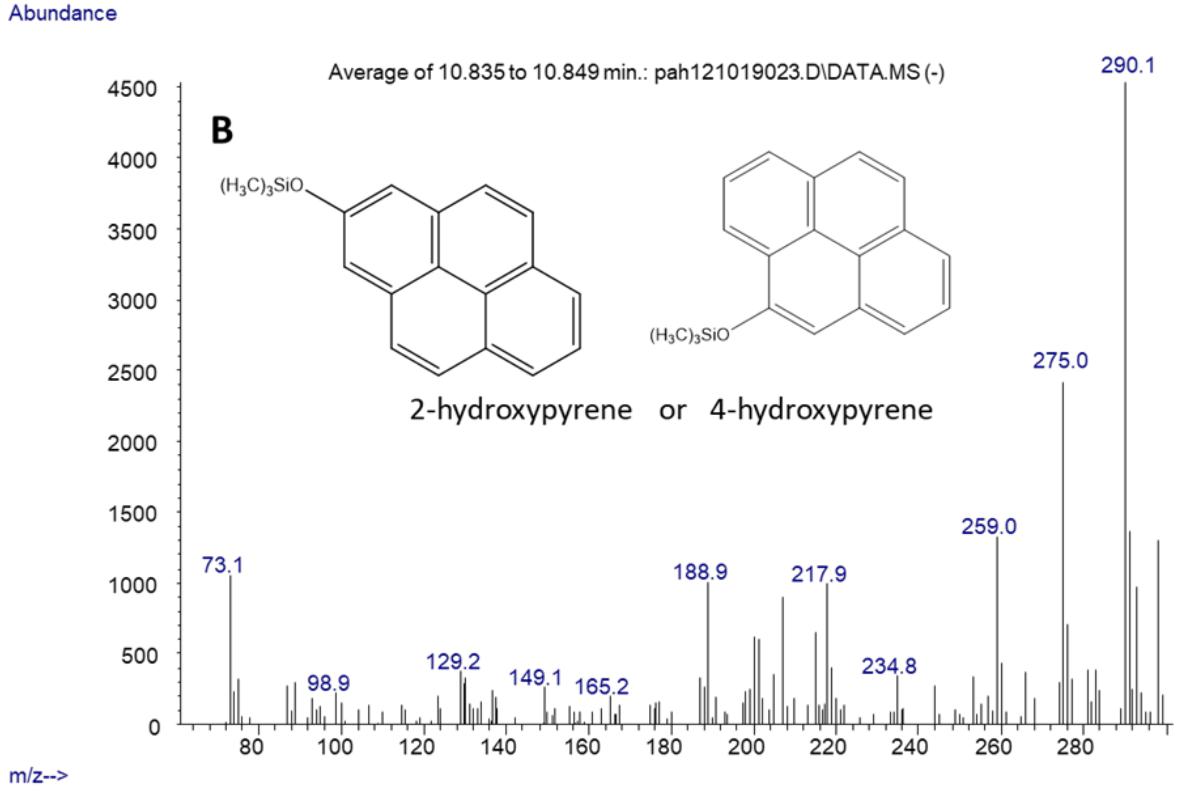

Supplemental Figure 2: Electron ionization mass spectra of silylated **A)** 1-hydroxypyrene and **B)** a hydroxy pyrene metabolite isolated from a pyrene mycobacterium incubation.

Supplement table 2

PAH metabolizing ability in known strains

Oral microorganism	PAH metabolism	Soil or tobacco leaf	Human commensal
C. albicans,	-	-	+
Acinetobacter junii,	+	+	-
Acinetobacter baumannii,	+	+	-
Agrobactrium tumerfaciens,	+	+	-
Actinomyces,	*?*	+	+
Bacillus pumillus,	+	+	-
Bacillus subtilis,	+	+	-
Kocuria rhizophila,	+	+	+
Rhodococcus,	+	+	+
Staphylococcus epidermidis,	+	+	+
Staphylococcus	+	+	+
Micrococcus Luteus	+	+	+
Kingella	*?*	-	+
